# Improving Patient Well-Being as a Broader Perspective in Dentistry

**DOI:** 10.1016/j.identj.2023.05.005

**Published:** 2023-06-19

**Authors:** Arden Mills, Yuli Berlin-Broner, Liran Levin

**Affiliations:** a Faculty of Pharmacy and Pharmaceutical Sciences, University of Alberta, Edmonton, Alberta, Canada; b Faculty of Medicine and Dentistry, University of Alberta, Edmonton, Alberta, Canada

**Keywords:** Oral health, Quality of life, Screening, Chronic disease, Noncommunicable diseases

## Abstract

Patient well-being encompasses the physical, mental, psychological, and social health of an individual. To adequately treat an individual and increase their quality of life, whole-person, patient-centred care needs to be utilised. This review aims to concisely summarise ways to improve patients’ well-being through and in dentistry. Oral health is tied to one's quality of life through oral function, overall health, self-perception, social acceptance, and social interaction. These relationships demonstrate the importance of utilising oral health to increase patient quality of life, unify health professions in patient treatment, use preventative medicine, and empower patients about their health. To do so, the dental profession can increase the scope of practice to provide preventative health screening and education on general health, have more open communication, collaborate with other health care professionals, and have broader consultations. This will allow for better continuity of care and shift the focus of treatment to the whole person instead of a symptom. Whilst there are barriers that need to be resolved and cost feasibility requires more exploration, the potential benefit to patients is apparent.

## Introduction

*Health* is an evolving construct that has a subjective meaning. In general, the term health has expanded in its definition to be more inclusive and has transitioned from just physical health to overall well-being. This shift allows health to encompass total body well-being and includes physical, mental, emotional, social, and spiritual components. To align with the multidimensional nature health has gained, the World Health Organization (WHO) created a unified definition of health in 1948: “a state of complete physical, mental, and social well-being and not merely the absence of disease or infirmity.”^1^ This demonstrated the importance of other aspects of health and created a unified definition; necessary for cohesion in practice, policy, and health services.^2^ Although this definition is believed to be too narrow in some respects, no other definition has received a wide range of consensus.^2^

Individual *well-being* includes a positive state of mind, functioning, satisfaction, and fulfillment in life.^3^ More specifically, well-being integrates all aspects of health, such as social, physical, and psychological health, with additional factors like life satisfaction, development, and activity.^3^ This shows that overall health is crucial in developing or determining one's well-being. Additionally, this demonstrates why health is subjective. Individuals have different views, priorities, and attitudes, which alter what health means and create internal satisfaction standards. Because individual well-being is subjective and self-reported measures can vary from objective measures, it is preferable to look at both the subjective and objective indicators^3^ when providing care.

As the definition of health has evolved towards encompassing overall well-being, it is essential to look at health disciplines similarly. In dentistry, for example, poor oral health impacts not only physical health but also mental and social health.^4^ Therefore, this review aims to concisely summarise ways to improve patients’ well-being through and in dentistry.

## Oral health, well-being, and quality of life

**Fig. 1:**
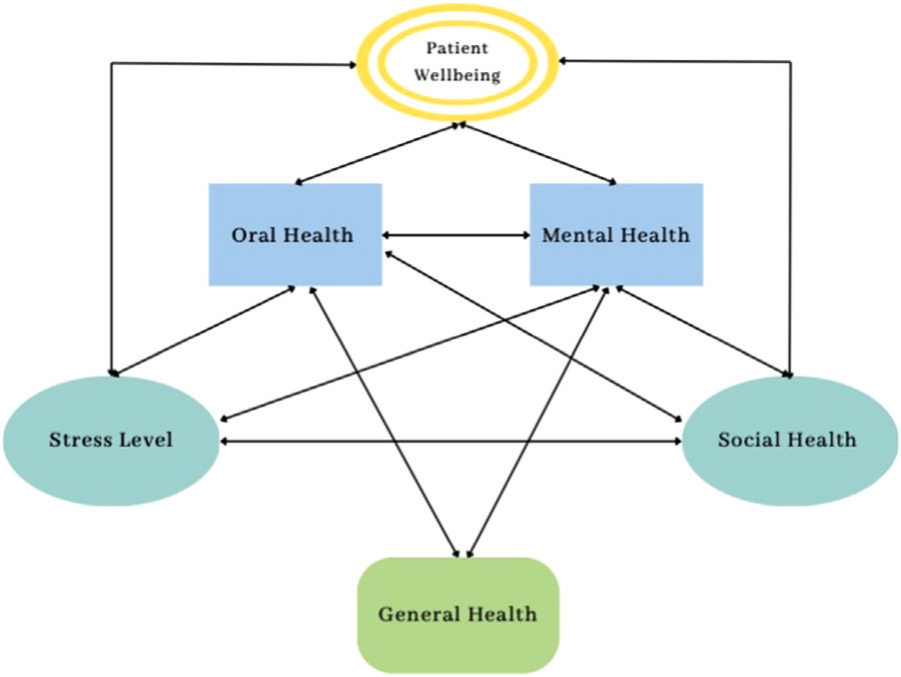
Patient well-being is interconnected with oral health, mental health, social health, stress, and general health. All aspects need to be considered when assessing well-being.

Oral health is tied to one's quality of life through oral function, overall health, self-perception, social acceptance, and social interaction (Figure 1). For example, dental diseases such as dental caries can cause pain, impaired chewing, reduced appetite, sleep disturbances, and reduced daily performance.^5^ Dental diseases can also lead to edentulism, impacting speech and facial shape, which affects the psychological well-being of individuals^6^ and confidence during social interactions, self-perception, stress levels, feelings of depression, isolation,^7^ and frustration.

The link between social belonging and dental health has historical ties, which include teeth alterations (shaping or filing, embedding jewels, bleaching, capping, and orthodontics) to indicate status, power, or attractiveness.^8^ These compounding effects on an individual's well-being can lead to financial burden through costly treatments for oral health and possible resulting comorbidities, medications, and other types of therapies. For instance, periodontal disease and its treatment impacts glycaemic control in diabetes.^9^ Additionally, diabetes requires pharmacologic treatment with insulin, with the adverse effect of weight gain, which may require further intervention and management. These conditions together can result in stress and create a need for psychological intervention. all of which create financial burden. Overall, the effects of oral conditions are far-reaching and can impact one's quality of life in many regards.

Halitosis and xerostomia can further illustrate the relationship between oral health and one's quality of life. Halitosis is highly prevalent and can affect up to 50% of the population.^10^ More than 90% of the time, halitosis can originate from poor oral hygiene, tongue coatings, xerostomia, periodontal disease, trapped food debris, and deep carious lesions.^11^ However, it can also arise from respiratory and gastrointestinal systems and be a symptom of menstruation, hematologic diseases, or medications.^10^ This demonstrates that the aetiology of a dental condition may be complex and shows the bidirectional nature of overall health.

Those affected by conditions such as halitosis and xerostomia are affected psychologically and socially. Affected individuals may be more apprehensive of social intimacy, decrease their overall social activity, and be predisposed to anxiety and other mental health disorders.^12^ Because halitosis can have various causes, multidisciplinary treatment of dentists and psychological caregivers is recommended for its management.^12^ This also solidifies the importance of dentists having basic mental health training and knowledge of accessible supports outside the dental field to better support their patients.

The interconnected nature of the body is bidirectional, meaning that nondental conditions can also impact oral health. For example, uncontrolled diabetes^13^ and depression (with use of antidepressant medications)^14^ can lead to xerostomia. The resulting xerostomia can increase the risk of dental caries; make eating, swallowing, and speaking difficult and unpleasant; and cause a sore throat or teeth sensitivity. Overall, this can affect dietary intake, physical health, and quality of life.^14^^,^^15^

The mouth has even been described as a mirror of the health in the rest of the body^16^ and can be a social indicator. Some social indicators include stress levels indicated by bruxism, overall health status, and oral health literacy.^17^ This mirror reflects that oral examination can aid in detecting many general health problems, such as nutritional deficiencies, microbial infections, immune disorders, cancer, and systemic diseases.^16^^,^^18^ Amongst the systemic diseases are HIV,^18^ cardiovascular diseases,^15^ diabetes,^19^^,^^20^ Alzheimer's disease,^21^ rheumatoid arthritis,^22^ cancer,^23^ and respiratory diseases.^24^ This reinforces the idea that oral health is closely tied to overall health.

Cultural, religious, spiritual, and personal considerations should be accounted for when assessing oral health and quality of life. For example, some patients may believe that dental pain is an inevitable part of an illness or a punishment^25^ or may not believe in western medicine. These perspectives must be respected when providing care and, if possible, should be integrated into treatment plans. This builds patient relationships and helps patients achieve a good quality of life according to their own standards.

## Treatment and improvement of patient well-being in the dental setting

### Preventative screening

Because oral health is vital in maintaining good overall health, there has been discussion about widening the scope of dentistry. This means that dental professionals would take a greater part in patients’ primary care. For example, patients could be screened for prediabetes and diabetes at visits,^26^ taking preventative action to reduce morbidity and mortality.^27^ This opportunistic screening could help identify the estimated 11.2% of patients with type 2 diabetes mellitus and 47.4% of patients with prediabetes who visit the dentist and are not yet diagnosed.^26^^,^^28^ Screening in a dental setting has the potential to improve patients’ care and well-being, especially because, for some, dental visits may be their most frequent or only contact with the health care system.^27^ Opportunistic screening of general health is also well perceived by dentists, patients, and physicians.^29^

Many of the screenings and interventions suggested have been for conditions directly or indirectly related to oral health. For example, monitoring for hypertension by measuring blood pressure during appointments has been proposed. This provides the potential to increase prevention, early detection, and treatment of resulting diseases, including heart disease, stroke, and kidney disease.^30^ However, when blood pressure screening procedures are implemented, white coat hypertension should be considered^31^; this is especially important because dental anxiety is common. Clinical studies have demonstrated that dental offices successfully detect patients at risk for or with hypertension and can influence patients receiving further care from general practitioners and pharmacists.^30^^,^^32^ This promotes a better continuity of care for all parties. However, cooperation between the fields will be essential to monitor patients and maintain accurate patient information successfully. Implementing a standardised database to solve this has yet to be explored.

Screening for infectious diseases, such as HIV, can be performed to provide more accessible testing.^33^ The test includes a rapid screening method which could be effectively incorporated into dental visits.^33^^,^^34^ This can increase the early diagnosis of HIV, since, for example, 1 in 4 individuals in Canada who are infected with HIV, are unaware of that.^34^ However, there is conflicting information on the barriers to implementing screening, professional concerns, and practice difficulties. HIV screening is said to be well accepted by patients,^32^ but there is public and professional debate about its relevance to dentistry.^33^^,^^34^ There are also questions about billing and providing patient support services, especially the initiative is not publicly funded. One study reported that insufficient reimbursement was a barrier to implementation.^35^ There is also a worry about being labelled as “HIV clinics.”^34^ Other concerns include patient privacy, preparing staff, ensuring continuing care with general practitioners, and support from dental organisations and insurance agencies.^34^^,^^35^ Overall, HIV screening has the potential to be beneficial to the public and impact societal well-being by aiding in the HIV epidemic; however, more information and direction are required before it can be properly implemented in the field.^36^

### Care continuity and awareness

Dental practitioners have the potential to assist with vaccinations and vaccination education. In selected countries, dentists are allowed to administer certain vaccines.^37^^,^^38^ The strain on workforce capacity during the coronavirus disease of 2019 (COVID-19) has helped influence the push to add vaccines into practice. However, even though dentists have been aiding frontline responses for over a decade,^39^ a Fédération Dentaire Internationale (FDI) survey revealed that two-thirds of countries had not permitted dentists to administer the COVID-19 vaccine.^40^ Permittance of providing vaccinations beyond that of the COVID-19 and influenza vaccines is yet to be passed in most regions. Whilst further training, education, and support will be required to ensure that all dental professionals can competently counsel and support patients, they can provide a larger platform for vaccine education^41^ and assist with maintaining more up-to-date records and boosters.

During emergencies, dental professionals can be a vital resource in ensuring patient continuity of care and can help compensate for the increased demand on medical resources and need for information. Some legislation recognises the importance of dental first responders and legalises scope-of-practice adjustments during emergencies.^42^ Roles dental professionals have assisted with include providing assistance to physicians in general treatment and patient monitoring, online counselling and prescribing, distributing medical supplies, evacuating and transferring patients, triage, specimen collection, and radiologic diagnosis.^42^ Additionally, dental professionals serve as an important source of health information and can decrease the spread of misinformation and disinformation during emergencies by educating the public. This could be especially impactful because dentists have professional relationships with their patients which include trust. In emergency planning, policymakers should consider the role dental professionals play in crisis management and professional associations should prepare professionals with continuing education classes that can be taken to fulfill yearly credits.

Dental professionals should also be aware of the One Health Initiative published by the Centers for Disease Control and Prevention in 2009. This initiative utilises a collaborative, multisectoral, and transdisciplinary approach to facilitate optimal health in individuals.^43^ One Health recognises that the health of humans is closely intertwined with that of animals the environments they share. They specifically focus on zoonotic diseases and emerging infectious diseases, preventing zoonoses, pandemic preparedness, water contamination, and global health security.^1^ Oral health professionals can broaden their scope of practice by being aware about and educating on microbial resistance and emerging diseases. For example, Lyme disease has been associated with chronic orofacial pain and facial paralysis from temporomandibular disorders.^44^ Additionally, saliva is a mechanism of transmission of many contagious conditions including COVID-19. By staying up-to-date on zoonotic and infectious diseases that are related to oral health, dental professionals will play a major role in increasing overall patient well-being by educating to prevent disease and identifying oral manifestations of these disease before detrimental impacts to health occur.^44^

To promote and protect the overall health of patients, incorporation of green dentistry into private practices should be considered. This coincides with the One Health Initiative, which indicates that environmental health is closely tied to human health.^44^ Implementing green dentistry would increase patient well-being indirectly by reducing environmental pollution. Types of dental pollution include general office waste from items like single-use plastics and wastes from procedures like biomedical wastes, lead, mercury, and silver. Practice owners should consider ways to reduce these types of wastes from their practices and how they are disposed.

### Patient lifestyle and history

Comprehensive dental care should include keeping track of habits and life changes or challenges. Some of these include pregnancy, nail-biting, bruxism, smoking, alcohol intake, sleep apnea, diet, and psychological conditions. By having broader consultations, these lifestyle aspects can be monitored and allow for referrals to other health professionals, improving patient well-being. This interprofessional communication is essential because there is often overlap in the condition itself. For example, bruxism can result from stress, indicating a deeper psychological problem. An intraoral device, such as a nightguard, can prevent tooth injury and the consequences of bruxism^45^ but would not solve the fundamental problem. Furthermore, smoking and caffeine intake can be contributing factors.^45^

Pregnant individuals should be educated on the relationship between oral health and pregnancy and have continuing dental care before, during, and after delivery. This is because pregnant individuals may be at an increased risk for periodontal disease, which has been associated with poor pregnancy outcomes.^46^ These outcomes include preterm delivery or having a baby with a low birth weight^47^ or preeclampsyia.^46^ Although some studies disagree with this relationship,^48^ risks should be minimised. In addition, pregnancy symptoms such as morning sickness and cravings also increase the risk of oral disease.^47^ This means that oral care is essential to ensure the health and well-being of both the mother and baby.

A comprehensive medical history should include the patient's history of smoking tobacco, cannabis, or any other substance as well as vaping. This will allow dental professionals to promote good oral health habits and assist in interventions. Specifically, dental professionals can assist in tobacco cessation promotion, monitoring, and encouragement or support. The benefit from interventions by dental professionals is supported by an increased likelihood of tobacco cessation compared to no intervention.^49^ There is conflicting evidence on whether behavioural support alone significantly changes quit rates, but increased contact with those attempting to quit smoking positively influences tobacco cessation success.^49^^,^^50^^,^^51^ Dental professionals also have the unique advantage of being able to recognise signs of smoking on the oral mucosa which can prompt discussions.

Tobacco cessation discussions can be based around the 5A's and 5R's models to effectively assess and discuss smoking cessation with patients.^51^ Discussions should inform patients on adverse health outcomes, utilise visuals and images to show patients the effect of smoking on their oral health, and offer strategies to begin a cessation attempt and control cravings. Dental professionals should collaborate with general practitioners and pharmacists on cessation attempts to increase follow-up and care continuity. Psychological support should be considered as nicotine withdrawal can be accompanied by depression, and nicotine addiction is prevalent in those with mental health disorders. Because smoking tobacco is a leading cause of preventable disease and death,^52^ promoting tobacco cessation through dental interventions can increase quality of life and successful smoking cessation attempts. Additionally, since smoking cannabis and vaping have become more prevalent, risks and potential risks should also be discussed as information becomes available.

### Collaboration

Although dentists feel that dietary advice is valuable, it is not utilised often enough.^53^ Providing more comprehensive care could include expanding dental teams to include dieticians^54^ or referring patients to dieticians.^55^ This would allow patients to be counselled and educated on their diet to prevent or control related diseases such as caries and diabetes.^55^ This would benefit patients by ensuring that adequate education is provided instead of the limited and brief dietary advice that has been reported.^53^ Additionally, a dietician has expertise in finding alternative foods and creating nutrition programmes to help facilitate behavioural changes in dietary habits.^56^ Preliminary evidence demonstrates that dieticians in dental settings can help patients change dietary behaviours.^56^ This can potentially provide patients with more resources to help alter their habits.

### Increased emphasis on preventive measures

Utilising preventative dentistry can empower patients about their health and increase their well-being and quality of life. For instance, patient education and awareness on common oral diseases such as dental caries and periodontal disease will assist patients in taking individual preventative measures and better control existing disease to aid in long-term health.^57^ This includes understanding the importance of oral home care, the use and appropriate selection of oral health products, periodic recall appointments, and preventative lifestyle measures.^58^ These measures have been shown to decrease the frequency of carious lesions,^58^ which has a direct impact on patient well-being. At dental appointments, oral health professionals should incorporate time to identify risk factors for oral disease and subsequently help patients with modifying risk factors.^59^ Thus, dental professionals should take an active role^59^ in preventing dental disease as opposed to passively treating the resulting diseases. It is in the patient's best interest that oral health practitioners perform both roles during care.

Dental trauma should also be included in preventative dentistry. Whilst sports provide outlets for exercise, mental exhaustion or stress, social interaction, and much more, they can increase the risk of dental trauma occurring.^60^, ^61^ Mouthguards, for example, significantly reduce the risk of orofacial injuries during sports and some sports have even mandated their use.^62^, ^63^ Mouthguards protect the teeth but were also reported to reduce the chance of subarachnoid haematoma and may protect against concussion.^62^ The benefits of mouthguard use are clear and dentist motivation is vital in athletes using them, along with compliance.^62^, ^64^

Successful preventative measures require patient compliance, which can be difficult to achieve.^65^ Barriers to compliance should be addressed, as in utilising teledentistry^17^ or care calls to check in if the patient is unable to attend in person at the frequency desired due to geographical or other social constraints (eg, time off work). This is an accommodation to reduce stress and increase continuity but is not a substitute for the complete absence of in-person care.

Preventative measures reduce the likelihood of the development of a chronic oral disease, worsening comorbidities early in life, and the occurrence of dental injury. These measures increase well-being and quality of life by preventing pain, cost, psychological insecurity, inconvenience, and the years lived with a chronic illness that is associated with dental and correlated diseases or conditions.

### Feasibility and cost

Preventing the development and progression of diseases and conditions is important to maintain a high level of well-being throughout society. By becoming aware of the bilateral relationships between oral health and systemic health, action can be taken by both patients and health care providers to improve patient well-being. There are minimal studies exploring the feasibility of co-integrated care^66^ and costs associated with these changes to practice. However, it has been suggested that whilst there are substantial hurdles to integrated care and profit will decrease from private practice, net revenue will remain positive.^66^ This means that utilising integrated care could decrease profits in dentistry private practices, but not to an extent that would make it monetarily unfeasible for owners. Implementing diabetic screening, for instance, can be cost-effective.^67^ Implicit benefits should also be considered, including increased patient well-being and better continuity of care. Preventative and interventional oral care has the potential to lower health care costs and increase general health outcomes.^68^^,^^69^ These economic evaluations must be examined further to better inform resource allocation and decision-making. One suggestion has been to include generic health outcome measures in the evaluations.^70^

## Recommendations

Dental professionals can increase patient well-being through dental treatment by incorporating greater preventative screening into routine visits, including for diabetes, high blood pressure, diet, and habits (eg, smoking and teeth clenching). This will allow for better monitoring of chronic diseases and provide more patient education opportunities to increase continuity of care. Additionally, by educating patients more often on their conditions and increasing awareness of how habits impact health, patients will be better equipped to advocate for and manage their health. Patient education can also include the relation of infectious or zoonotic diseases on oral health and the resulting implications to general health. Other preventative measures dental professionals can implement include incorporating green dentistry into their practice.

Improving communication amongst dental providers, patients, and other health disciplines will be crucial for the effectiveness of preventative screening and as another tool to increase well-being. Developing open conversations with patients and understanding patients’ needs, expectations, and cultural differences will help facilitate this. Additionally, better and more frequent communication is highly important for interdisciplinary and interprofessional collaboration in patient treatment. Healthcare providers will need to know where and when to refer a patient to seek specialised care, whether from a dietician, psychologist, or other health professionals. This will aid in the patients’ continuity of care and further increase well-being by ensuring all patient health needs are met and cared for appropriately.

**Fig. 2:**
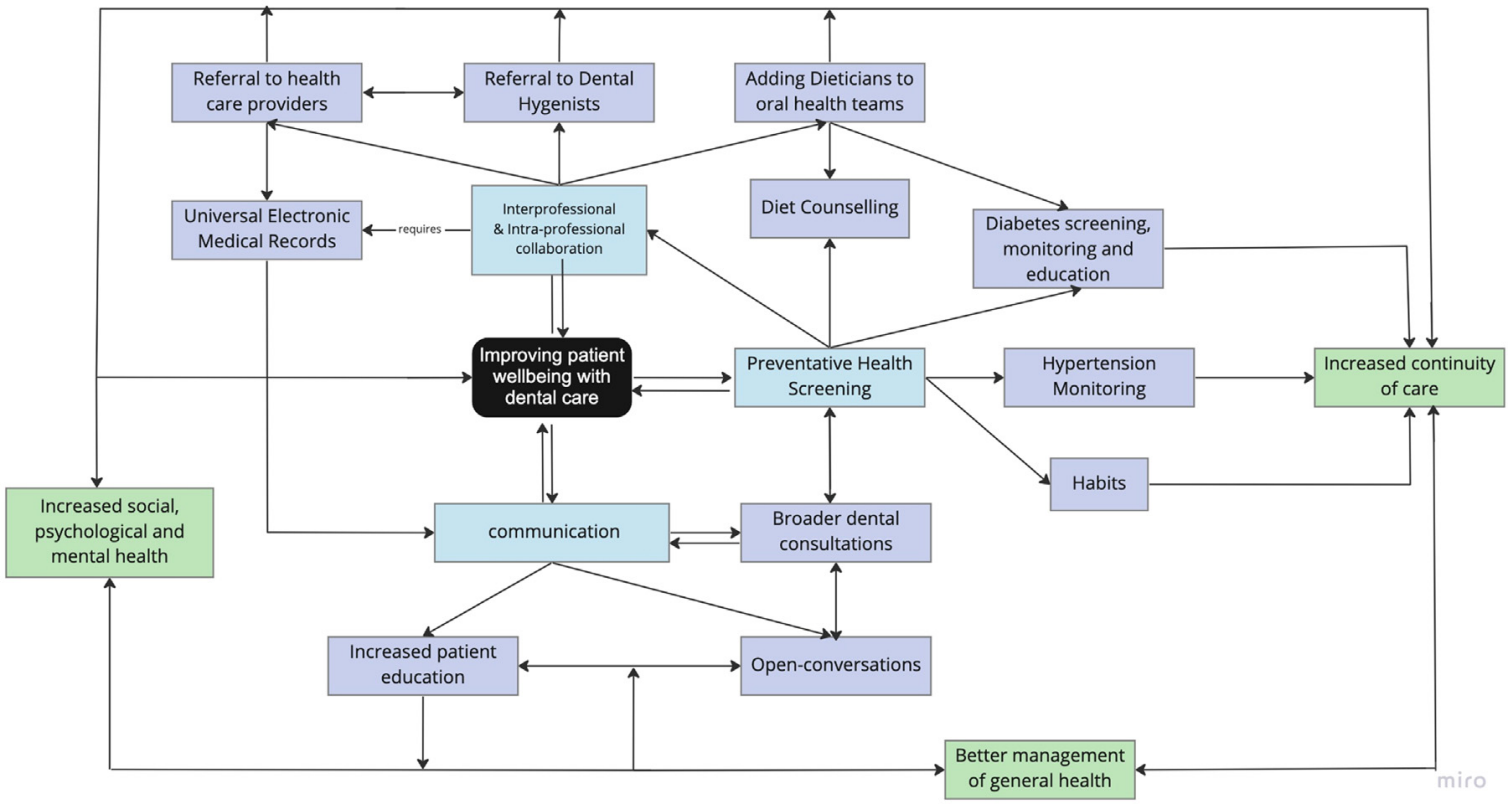
Improving patient well-being in dental care with communication, preventative screening, and inter- and intraprofessional collaboration. This will facilitate an increase in the continuity of care, provide better management of general health, and contribute to increased social, psychological, and mental health.

By incorporating these aspects, dental professionals will assist with managing general health, continuity of care, and increased social, psychological, and mental health. These aspects are intertwined and will help dental professionals treat the whole individual to better patient well-being (Figure 2).

### Incorporation into practice: call for educators

To incorporate these aspects into dental practice, dental professionals will need to have access to appropriate resources for education and support. Dental schools should consider adding more comprehensive information on preventative screening, utilise comprehensive medical history assessments when teaching, provide more information about collaborative practice for continuity of care, and offer resources to find information or support with these activities. To educate those already practicing and provide continuing education, dental associations should consider providing continuing education credit courses. Associations should also consider ways to provide practicing dentists support with these activities and direction if needed. Dental assistant and hygiene programmes or associations should also look to incorporate these aspects into their curriculum.

## Conclusions

To improve patient well-being in dental care, preventative health screening, more open communication, greater patient education, integrated health care software, and interprofessional collaboration should be utilised. This will allow better continuity of care and shift the focus of treatment to the whole person instead of a symptom. Whilst there are barriers that need to be resolved and cost feasibility requires more exploration, the potential benefit to patients is apparent.

## Author contributions

All authors were involved in the design and development of the review. All contributed to the writing and approved the final version.

## Conflict of interest

None disclosed.
